# Next-generation sequencing for virus detection: covering all the bases

**DOI:** 10.1186/s12985-016-0539-x

**Published:** 2016-06-02

**Authors:** Marike Visser, Rachelle Bester, Johan T. Burger, Hans J. Maree

**Affiliations:** Agricultural Research Council, Infruitec-Nietvoorbij: Institute for Deciduous Fruit, Vines and Wine, Stellenbosch, South Africa; Department of Genetics, Stellenbosch University, Stellenbosch, South Africa

**Keywords:** CTV, Closterovirus, Genome coverage, GLRaV-3, Next-generation sequencing, Sequencing depth, Virus detection

## Abstract

**Background:**

The use of next-generation sequencing has become an established method for virus detection. Efficient study design for accurate detection relies on the optimal amount of data representing a significant portion of a virus genome.

**Findings:**

In this study, genome coverage at different sequencing depths was determined for a number of viruses, viroids, hosts and sequencing library types, using both read-mapping and *de novo* assembly-based approaches. The results highlighted the strength of ribo-depleted RNA and sRNA in obtaining saturated genome coverage with the least amount of data, while even though the poly(A)-selected RNA yielded virus-derived reads, it was insufficient to cover the complete genome of a non-polyadenylated virus. The ribo-depleted RNA data also outperformed the sRNA data in terms of the percentage of coverage that could be obtained particularly with the *de novo* assembled contigs.

**Conclusion:**

Our results suggest the use of ribo-depleted RNA in a *de novo* assembly-based approach for the detection of single-stranded RNA viruses. Furthermore, we suggest that sequencing one million reads will provide sufficient genome coverage specifically for closterovirus detection.

**Electronic supplementary material:**

The online version of this article (doi:10.1186/s12985-016-0539-x) contains supplementary material, which is available to authorized users.

## Findings

Next-generation sequencing (NGS) has proven to be a valuable tool for virus detection, discovery or diversity studies and has increased in popularity, while decreasing in cost. The percentage genome-wide coverage obtained, either through the mapping of reads or contigs (assembled reads) onto a reference genome, can serve as a form of virus detection. The confidence in a positive identification increases with greater coverage. Due to the variation in the number of reads associated with different genomic regions, an uneven coverage of the viral genome is often observed in RNA-Seq data. Variation in sequencing depth will, consequently, influence the percentage of genome coverage that can be obtained. It is therefore necessary to find the optimal amount of data needed to cover the complete or almost complete genome without generating an excess of sequence data. Therefore, the aim of this study was to illustrate the influence of sequencing depth on virus and viroid genome coverage, to provide a guideline for the number of reads required to offer maximum possible genome coverage.

In this study two viruses from the family *Closteroviridae*, were selected. They were known variants of *Grapevine leafroll-associated virus 3* (GLRaV-3) (variant group II) and *Citrus tristeza virus* (CTV) (genotype T3) that were graft-inoculated into *Vitis vinifera* (grapevine) and *Citrus paradisi* (grapefruit) plants, respectively. While the exact sequence information for the GLRaV-3 variant was available the genome of the specific CTV inoculum has not been sequenced. Along with the virus, the grapevine plants were additionally infected with *Hop stunt viroid* (HSVd) and the grapefruit plants with *Citrus dwarfing viroid* (CDVd). The viroid infections were first identified in the sequencing data. Phloem material was sampled from 12 grapevine and 6 grapefruit plants and total RNA was extracted, using a CTAB method adapted from Carra et al. [1]. RNA was sent for sequencing to Fasteris (Geneva, Switzerland). A small RNA (sRNA) library and a transcriptome library were prepared from each RNA extract and sequenced on an Illumina HiSeq instrument (1 × 50 for sRNA runs, 1 × 125 for grapevine and 2 × 125 for grapefruit transcriptomes). Poly(A)-selected and ribo-depleted RNA was used for the grapevine and grapefruit transcriptome sequencing, respectively. After adapter trimming, as well as quality filtering and trimming, sRNA reads with a minimum length of 18 nts and transcriptome reads with a minimum length of 20 nts were retained for further analyses.

To determine the minimum number of reads needed to cover a genome using read-mapping, subsets (ranging from 10,000 to 10 million reads) were extracted randomly, using a custom Python script, from each complete NGS dataset to simulate different sequencing depths. A 1000 bootstrap replication of each subset-size was randomly selected from the complete dataset to assign a measure of accuracy to the estimation of the mean genome coverage. The subsets of reads were mapped onto the genome of interest using Bowtie [2] for sRNA and Bowtie2 [3] for transcriptome data. Grapevine reads were mapped against GLRaV-3 isolate GP18 (Accession No. EU259806) and HSVd isolate 7139_HSVd_Sy (Accession No. KF007325) reference genomes. *Citrus tristeza virus* isolate T3 (Accession No. KC525952) and CDVd (Citrus viroid IIIc, Accession No. AF184149) reference genomes were used for citrus analysis. For the sRNA data a single mismatch per read was allowed while for the transcriptome data a single mismatch was allowed per seed of 20 nts. Following read-mapping, the genome coverage for each subset was determined by calculating the number of genome bases covered at least once. Heat maps were generated to show the variation in depth of coverage of genomic regions with increasing subset-size. The heat maps reflect the number of times (out of the 1000 replicates) a specific base was covered.

**Fig. 1:**
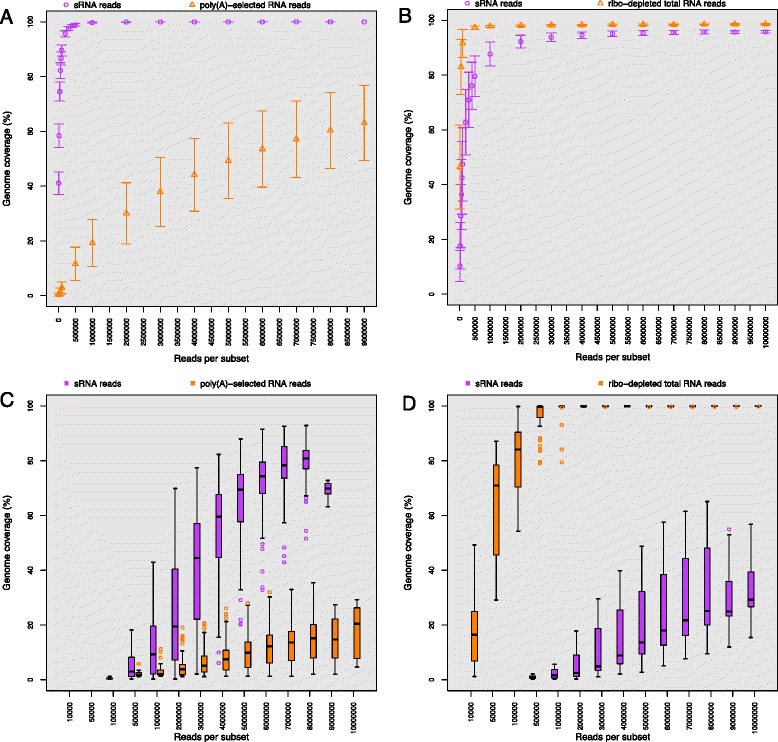
Average virus genome coverage obtained through read-mapping and *de novo* assembly. Graphs displaying the average and standard deviation of genome coverage obtained with read-mapping (1000 replications) of different subset-sizes of small (sRNA) reads and poly(A)-selected or ribo-depleted RNA reads onto the reference genomes of *Grapevine leafroll-associated virus 3* (GLRaV-3) (**a**) and *Citrus tristeza virus* (CTV) (**b**). Box-and-whisker plot displaying the data distribution of different subset-sizes of *de novo* assembled (10 replications) sRNA, poly(A)-selected or ribo-depleted RNA reads and subsequent mapping of the contigs onto the reference genomes of GLRaV-3 (**c**) and CTV (**d**)

**Fig. 2:**
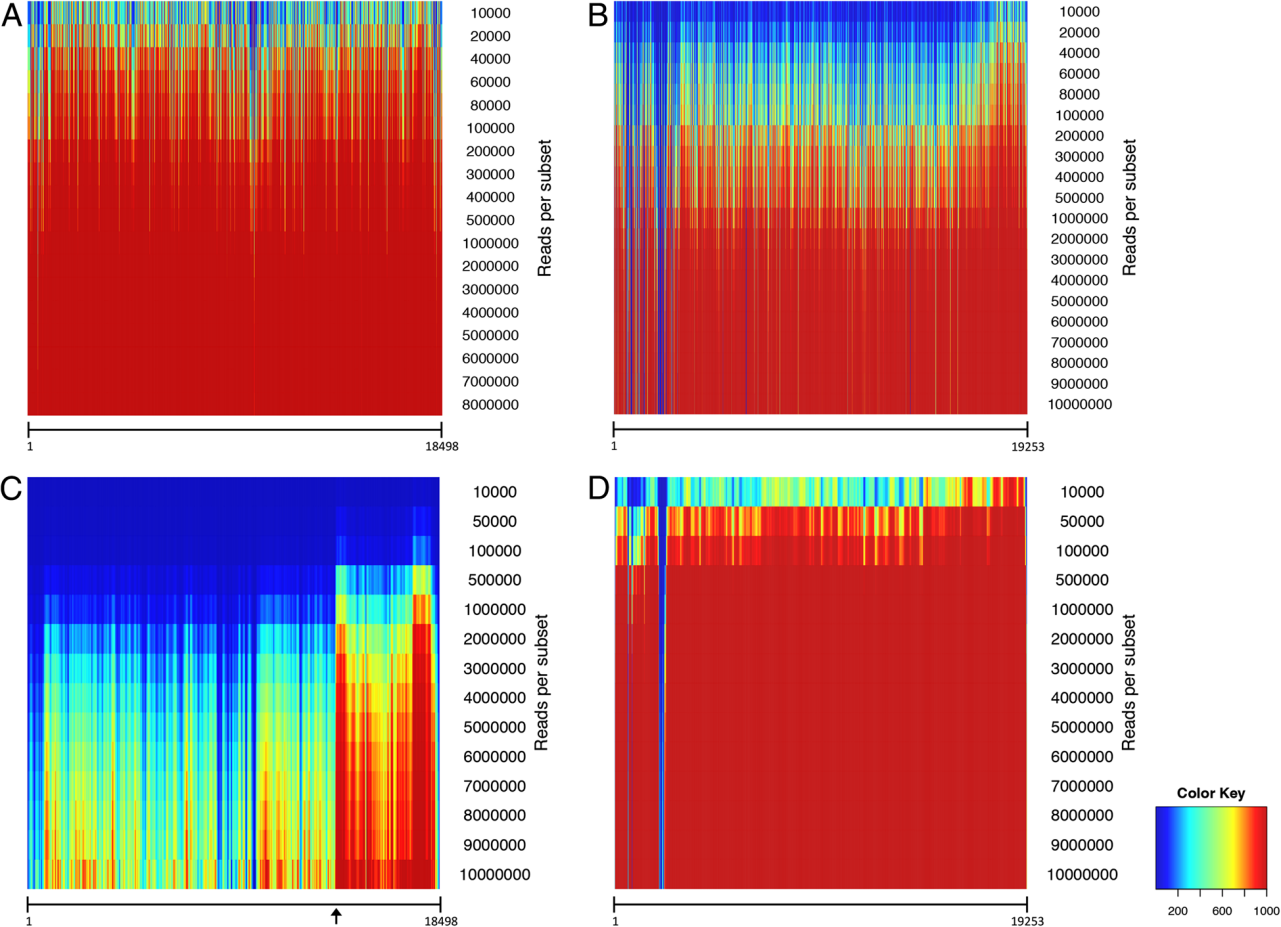
Average depth of virus genome coverage obtained through read-mapping. Heat maps displaying the average depth of coverage of each nucleotide along the virus genome (X-axis), obtained through read-mapping (1000 replications) of different subset-sizes of small (sRNA) (**a** and **b**) and poly(A)-selected (**c**) or ribo-depleted RNA (**d**) reads onto the reference genomes of *Grapevine leafroll-associated virus 3* (GLRaV-3) (**a** and **c**) and *Citrus tristeza virus* (CTV) (**b** and **d**). The start of the coat protein subgenomic RNA is indicated with an *arrow*

The results showed a low degree of virus genome coverage using transcriptome data generated from poly(A)-selected RNA for GLRaV-3 (Fig. 1a). At a maximum of nine million reads, the average genome coverage was below 65 %. This was in contrast to the almost complete genome coverage (99 %) reached with the sRNA data from approximately 0.5 million reads for the same reference genome. Neither of the virus species investigated have a polyadenylated genome. However, the 3′ bias of GLRaV-3 genome coverage obtained through sequencing of poly(A)-selected RNA may be ascribed, in part, to the expression of subgenomic RNAs (sgRNAs). In Fig. 2c a clear increase in coverage can be observed at around position 13,800 of the GLRaV-3 genome. This position corresponds to the sgRNA initiation point for GLRaV-3 sgRNAs of ORF6 (coat protein), which was found previously to be one of the most abundant sgRNA species [4].

Transcriptome data generated from ribo-depleted RNA reached saturation from approximately 0.5 million reads (250,000 pairs) and covered ~98 % of the complete CTV genome (Fig. 1b). The mapping of the sRNA data against the CTV genome produced a comparable result although the coverage seemed to reach saturation from approximately ~5 million reads, covering ~95 % of the genome. The grapefruit plants were graft-inoculated with a field sample infected with an unsequenced T3 variant. An area of the CTV genome, near the 5′ end, were either only slightly covered at the maximum sequencing depth or not covered at all (Fig. 2b, d). This illustrated an area of low homology between the inoculated T3 variant and its closest reference genome available on NCBI, which was used for mapping analysis.

The difference in sRNA genome coverage between the two virus species (Fig. 1a, b) could be explained by variation in the host-response to infection. The sRNA reads that could be mapped onto a virus genome represent sRNAs known as virus-derived small interfering RNAs (vsiRNAs). These vsiRNAs are generated by the host in response to virus infection [5] and can either be host [6, 7] or virus specific [8, 9]. A less severe or more targeted host response to CTV could explain the lower coverage of the CTV genome with sRNA data at lower sequencing depths (Figs. 1b and 2b), when compared to that of the GLRaV-3 sRNA data (Figs. 1a and 2a).

As could be expected for their considerably smaller genome size, less reads were required for the two viroids, compared to the viruses, to reach genome coverage saturation (Additional file 1: Figures S1A and S1B). Compared to GLRaV-3, the percentage of genome coverage by poly(A)-selected data was more similar to that of the sRNA data for HSVd in grapevine. Coverage of the genome termini seemed less efficient (Additional file 2: Figure S2). Mapping may be more complicated for viroids due to their circular nature and small genomes. This could influence read-mapping to the terminal areas of the linearised genome sequences since reads which cover both ends may not be able to map.

Reads were also *de novo* assembled into contigs and then mapped to the different reference genomes in order to calculate the genome coverage. Ten reference points, separated by one tenth of the data, were identified within each complete NGS dataset. Starting from each reference point, a subset of data was extracted that together formed the 10 replicates for a specific subset-size. Extraction was repeated from each reference point for the same subset-sizes (sequencing depths) as was used for the read-mapping analysis. Each subset of reads was then subjected to *de novo* assembly using CLC Genomics Workbench 8. A bubble size of 50, a word size of 20 and a minimum contig length of 200 were selected as *de novo* assembly parameters. The contigs were mapped onto the reference virus and viroid genomes to determine the coverage thereof using a mismatch cost of 2 and a similarity fraction of 0.95.

Resembling the results from the read-mapping analysis (Fig. 1a), a significantly higher GLRaV-3 genome coverage could be obtained with the *de novo* assembly of sRNA data than with poly(A)-selected data (Fig. 1c). The poly(A)-selected data did not cover more than 20 % of the genome, while the sRNA data could cover up to ~80 % of the genome. Also in agreement with the read-mapping results, the ribo-depleted data presented a higher CTV genome coverage (almost 100 % from as little as 0.5 million reads) than the sRNA data, which could not cover 30 % thereof (Fig. 1d). Except for the ribo-depleted data, significantly less of the virus genomes could be covered by contigs than through read-mapping. This was especially the case for the CTV genome with sRNA data (Fig. 1d). The lower genome coverage obtained through the *de novo* assembly approach can be expected due to the difficulty in assembling reads from large datasets, especially that of sRNA reads. Despite the lower coverage, similarity based searches using assembled contigs is still essential for detecting viruses when considering genome coverage as a form of detection, and cannot be avoided. In order to confirm the conclusion made from the *de novo* assemblies and to evaluate the impact that different assemblers will have on the genome coverage percentage, the CLC pipeline was compared to a Velvet/Burrows-Wheeler Alignment Tool (BWA) [10, 11] pipeline. VelvetOptimizer was used to select the best parameters for assembly after which the contigs were mapped to the respective reference genomes using BWA. Three subset sizes (100,000, 1,000,000 and 7,000,000) were compared. The velvet pipeline had a higher level of variation between samples and subset replications and failed to assemble a significant number of virus contigs with the 7,000,000 sRNA reads subsets for both GLRaV-3 and CTV (Additional file 3: Figure S3 and Additional file 4: Figure S4). However, both pipelines showed that ribo-depleted RNA produced better genome coverage for the detection of closteroviruses.

As with the read-mapping results, the *de novo* assembly results for HSVd corresponded to that of CDVd (Additional file 1: Figures S1C and S1D). Coverage saturation (~80 %) was reached with both transcriptome data-types, for HSVd and CDVd, with only 50,000 and 10,000 reads, respectively. A gradual increase in genome coverage was observed with the sRNA data nearing saturation (~80 %) from ~4 million reads for HSVd and ~2 million reads for CDVd. Viroid genome assembly and contig mapping may be influenced by their circular genomes, resulting in the lower coverage observed.

In spite of decreases in the cost of NGS, there is still a need to determine the optimal amount of data needed for accurate and reliable virus detection. The number of reads required for detection is influenced by the number of virus-derived reads in the data, the size of the virus genome and the complexity of the virome (not assessed here). Some viruses or viroids may be more represented in the data depending on (i) the degree of infection, which may be host and/or virus dependent, (ii) the plant response to infection in the case of sRNAs and (iii) the nature of the genome in the case of different library types (i.e., DNA, poly(A)-selected RNA, ribo-depleted RNA libraries). This study however focussed on two non-polyadenylated single-stranded RNA viruses. The genomes of these closteroviruses represent some of the largest plant-infecting RNA virus genomes (~19 kb), which along with the viroid comparisons covered the wide range of genome sizes that could influence detection.

We conclude that NGS data generated from ribo-depleted total RNA outcompeted data generated from poly(A)-selected RNA, potentially universally or at least for non-polyadenylated viruses such as closteroviruses. For some viruses, sRNA data may be equivalent to ribo-depleted total RNA data depending on the host response. For virus detection, we recommend sequencing ribo-depleted total RNA, followed by *de novo* assembly of the reads and alignment-based similarity searches for virus identification. Specifically for closteroviruses detection, we recommend obtaining one million high-quality reads. Overall, the results presented in this study serve as a guideline for selecting the number of NGS reads needed to represent a full virus (or viroid) genome.

## Additional files

Additional file 1: Figure S1. Average viroid genome coverage obtained through read-mapping and *de novo* assembly. Graphs displaying the average and standard deviation of genome coverage obtained with read-mapping (1000 replications) of different subset-sizes of small (sRNA) reads and poly(A)-selected or ribo-depleted RNA reads onto the reference genomes of *Hop stunt viroid* (HSVd) (A) and *Citrus dwarfing viroid* (CDVd) (B). Box-and-whisker plots displaying the data distribution of different subset-sizes of *de novo* assembled (10 replications) sRNA, poly(A)-selected or ribo-depleted RNA reads and subsequent mapping of the contigs onto the reference genomes of HSVd (C) and CDVd (D). (PDF 98 kb) Additional file 2: Figure S2. Average depth of viroid genome coverage obtained through read-mapping. Heat maps displaying the average depth of coverage of each nucleotide along the virus genome (X-axis), obtained through read-mapping (1000 replications) of different subset-sizes of small (sRNA) (A and B) and poly(A)-selected (C) or ribo-depleted RNA (D) reads onto the reference genomes of *Hop stunt viroid* (A and C) and *Citrus dwarfing viroid* (B and D). (PDF 2205 kb) Additional file 3: Figure S3. Average virus genome coverage obtained through *de novo* assemblies with CLC genomic workbench compared to Velvet. Box-and-whisker plot displaying the data distribution of different subset-sizes of *de novo* assembled (10 replications) sRNA (A, B), poly(A)-selected (C) or ribo-depleted RNA (D) reads and subsequent mapping of the contigs onto the reference genomes of GLRaV-3 (A, C) and CTV (B, D). (PDF 82 kb) Additional file 4: Figure S4. Average virus genome coverage obtained through *de novo* assemblies with CLC genomic workbench compared to Velvet. Box-and-whisker plot displaying the data distribution of different subset-sizes of *de novo* assembled (10 replications) sRNA (A, B), poly(A)-selected (C) or ribo-depleted RNA (D) reads and subsequent mapping of the contigs onto the reference genomes of GLRaV-3 (A, C) and CTV (B, D). For the 7,000,000 reads sRNA subsets, each replication was divided into 1,000,000 reads subsets for *de novo* assembly after which the contigs were pooled for mapping to the reference genome. (PDF 82 kb)

## Abbreviations

CDVd: *Citrus dwarfing viroid*
CTV: *Citrus tristeza virus*
GLRaV-3: *Grapevine leafroll-associated virus 3*
HSVd: *Hop stunt viroid*
NGS: next-generation sequencing
sgRNA: subgenomic RNA
sRNA: small RNA
vsiRNAs: virus-derived small interfering RNA
