# Supplementary figures and images for: Next-generation sequencing for virus detection: covering all the bases
Source: Virol J. 2016 Jun 2;13:85. doi: 10.1186/s12985-016-0539-x (PMC4890495; doi:10.1186/s12985-016-0539-x)

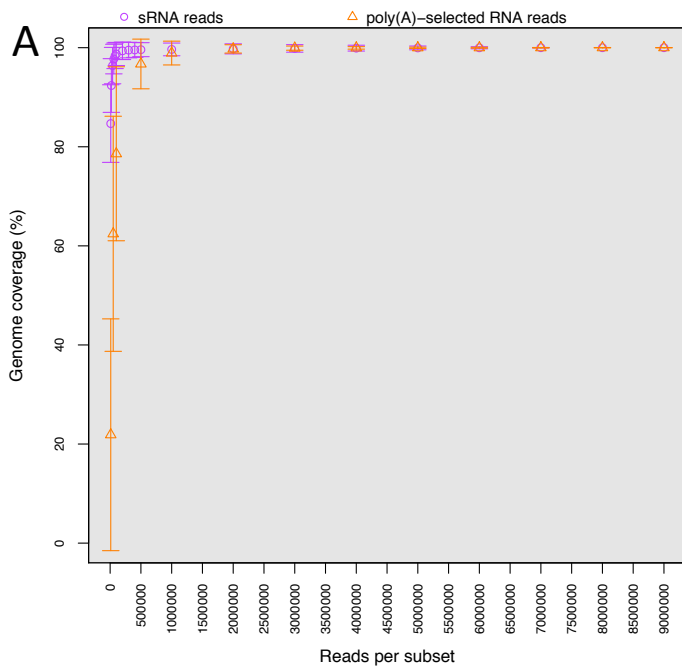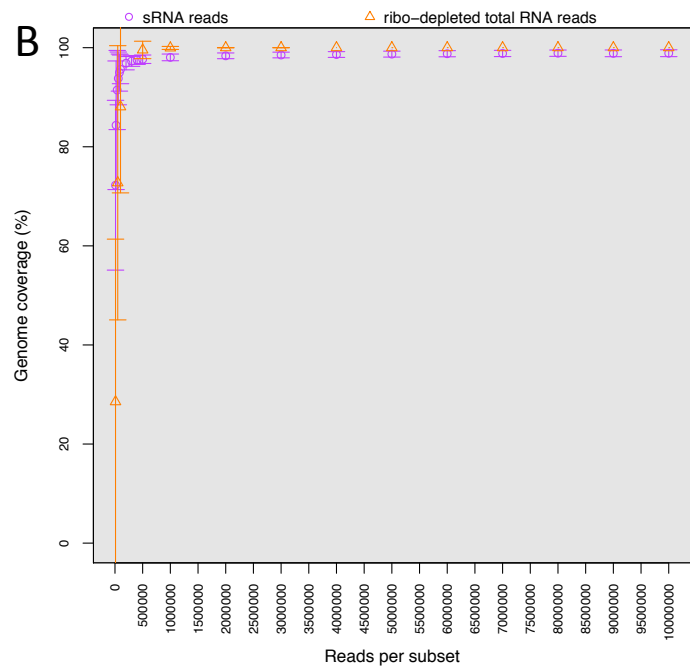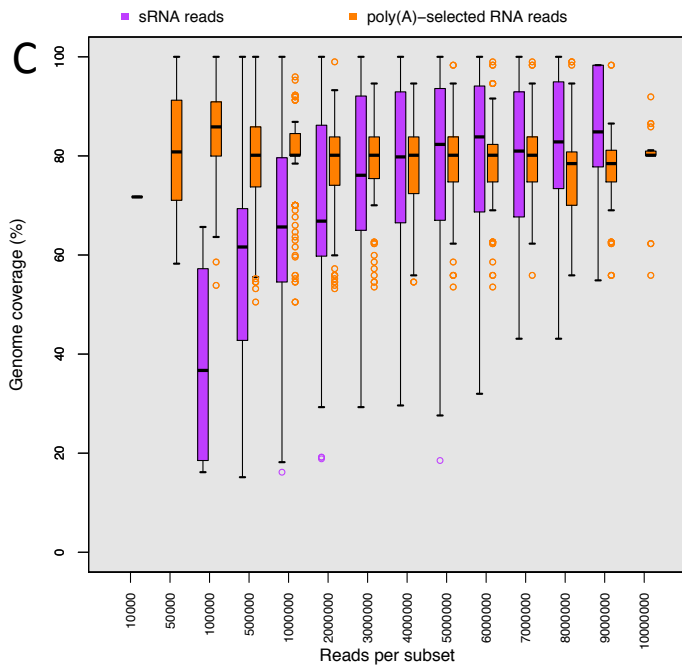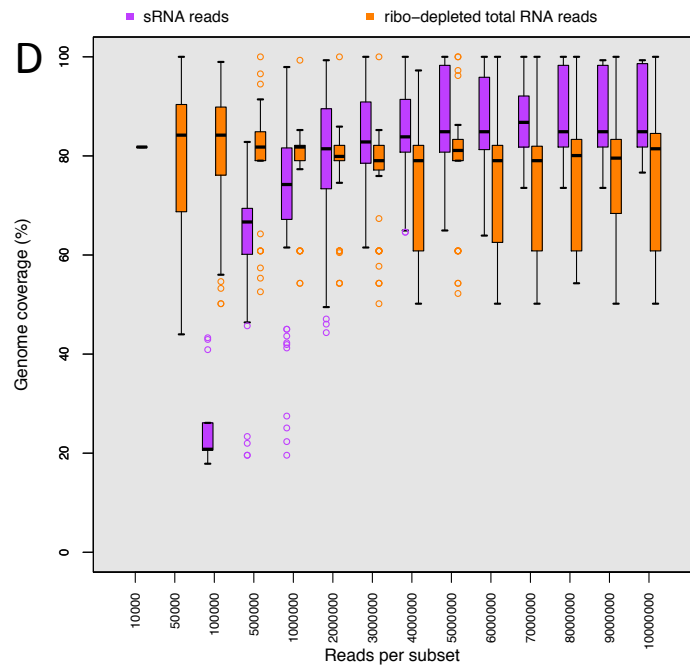

Supplement: Additional file 1: Figure S1. — Average viroid genome coverage obtained through read-mapping and de novo assembly. Graphs displaying the average and standard deviation of genome coverage obtained with read-mapping (1000 replications) of different subset-sizes of small (sRNA) reads and poly(A)-selected or ribo-depleted RNA reads onto the reference genomes of Hop stunt viroid (HSVd) (A) and Citrus dwarfing viroid (CDVd) (B). Box-and-whisker plots displaying the data distribution of different subset-sizes of de novo assembled (10 replications) sRNA, poly(A)-selected or ribo-depleted RNA reads and subsequent mapping of the contigs onto the reference genomes of HSVd (C) and CDVd (D). (PDF 98 kb) [file 12985_2016_539_MOESM1_ESM.pdf]

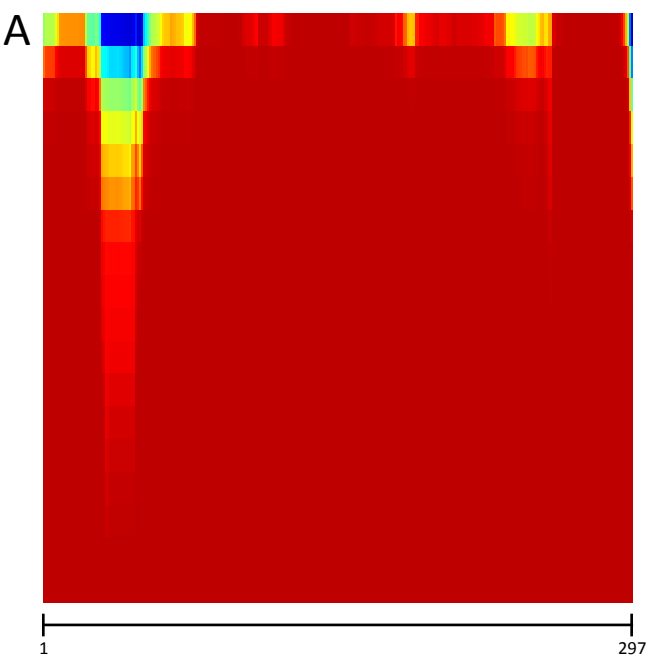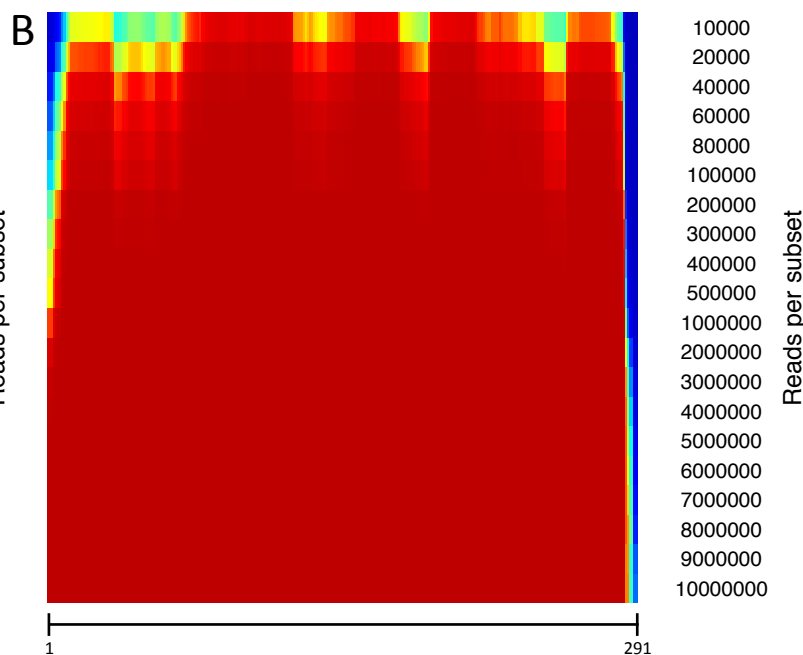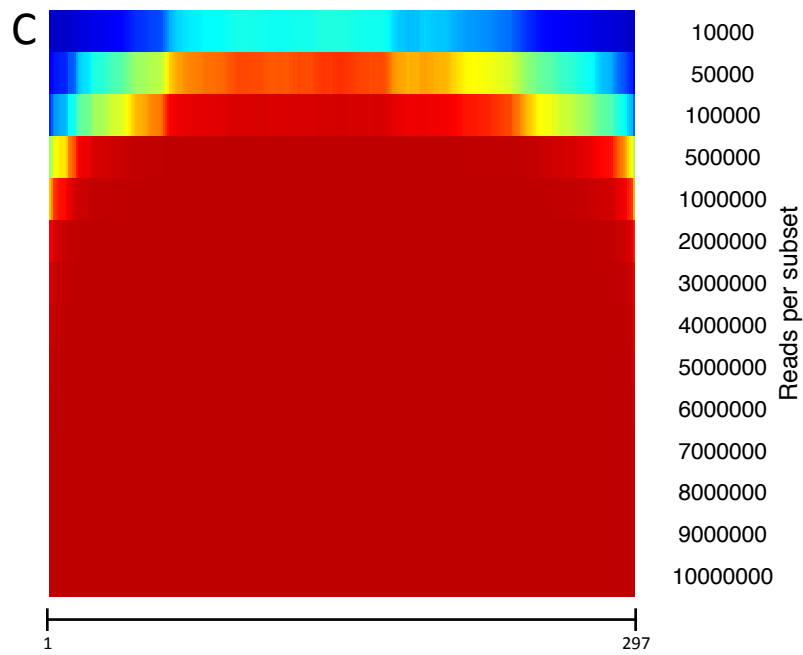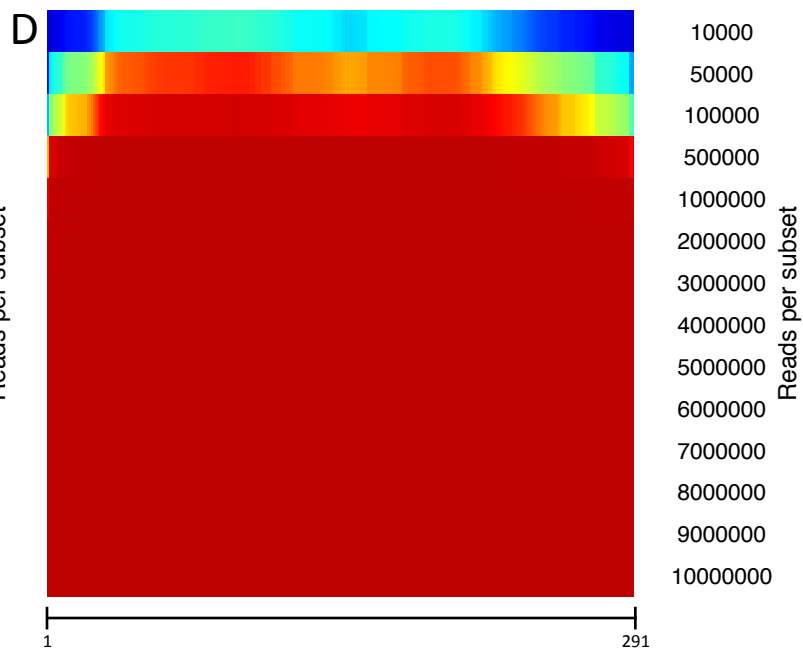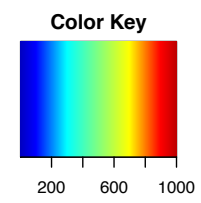

Supplement: Additional file 2: Figure S2. — Average depth of viroid genome coverage obtained through read-mapping. Heat maps displaying the average depth of coverage of each nucleotide along the virus genome (X-axis), obtained through read-mapping (1000 replications) of different subset-sizes of small (sRNA) (A and B) and poly(A)-selected (C) or ribo-depleted RNA (D) reads onto the reference genomes of Hop stunt viroid (A and C) and Citrus dwarfing viroid (B and D). (PDF 2205 kb) [file 12985_2016_539_MOESM2_ESM.pdf]

**A**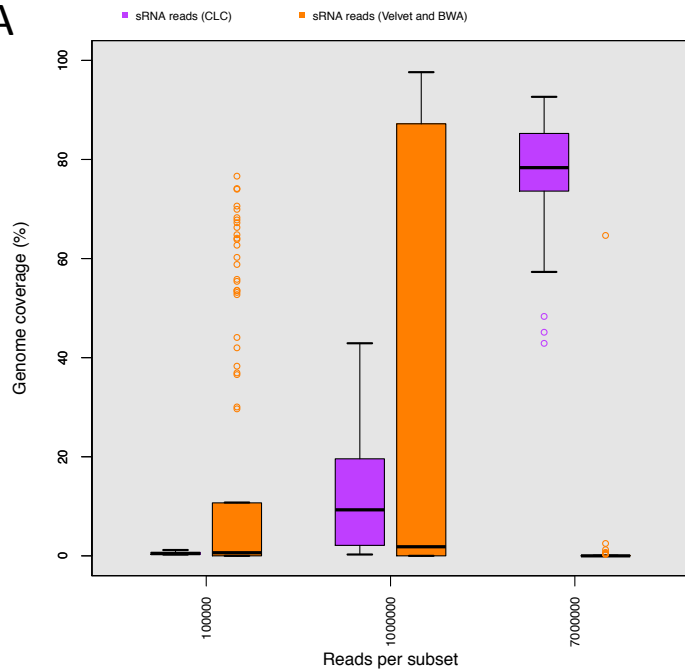**B**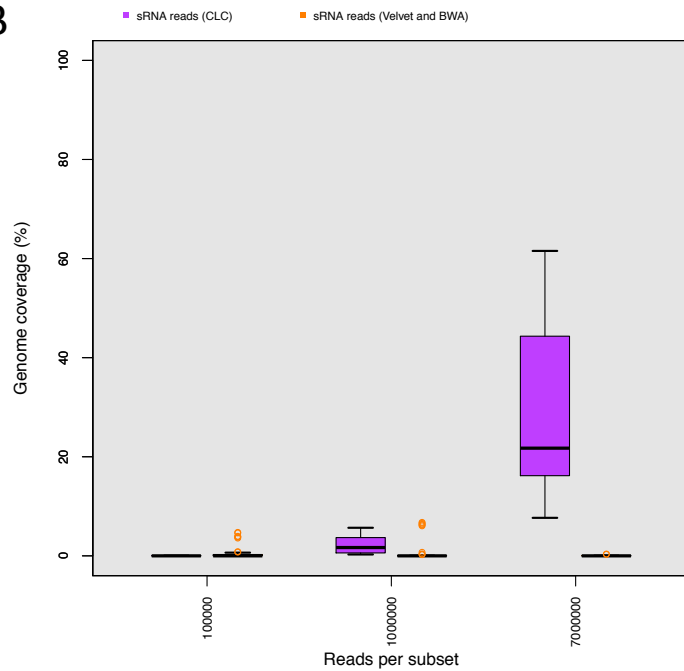**C**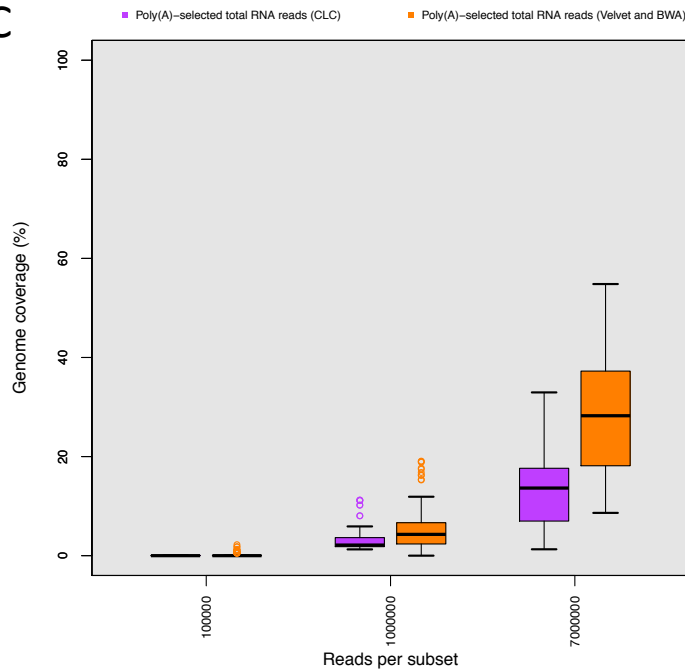**D**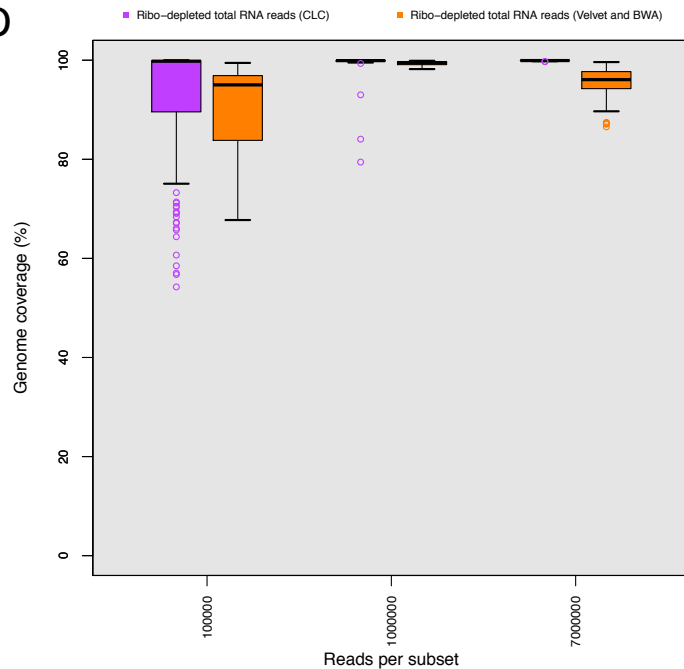

Supplement: Additional file 3: Figure S3. — Average virus genome coverage obtained through de novo assemblies with CLC genomic workbench compared to Velvet. Box-and-whisker plot displaying the data distribution of different subset-sizes of de novo assembled (10 replications) sRNA (A, B), poly(A)-selected (C) or ribo-depleted RNA (D) reads and subsequent mapping of the contigs onto the reference genomes of GLRaV-3 (A, C) and CTV (B, D). (PDF 82 kb) [file 12985_2016_539_MOESM3_ESM.pdf]

**A**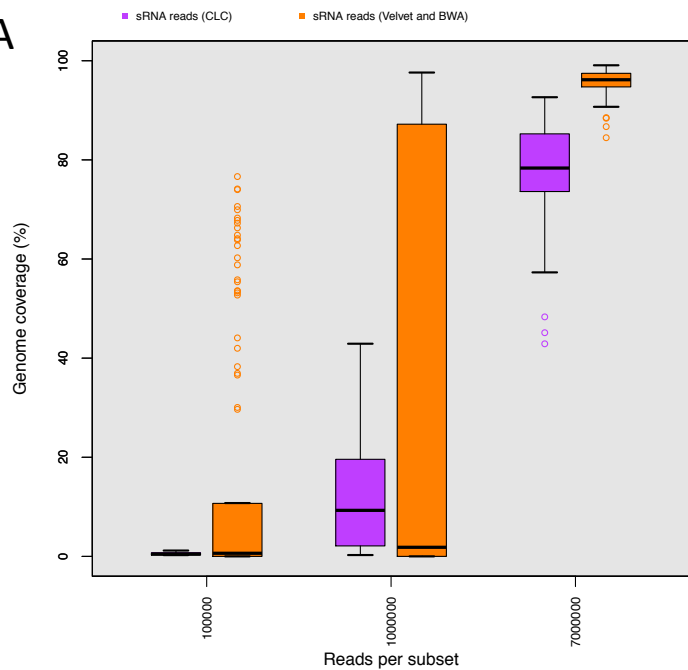**B**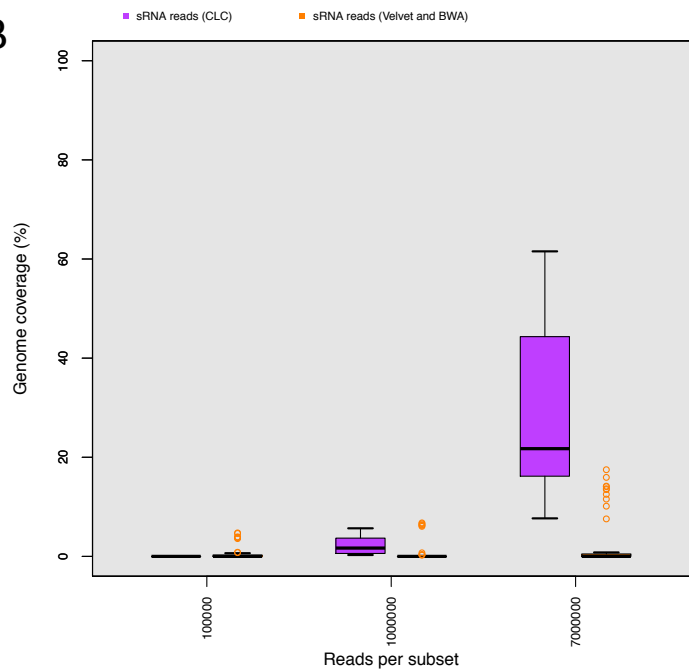**C**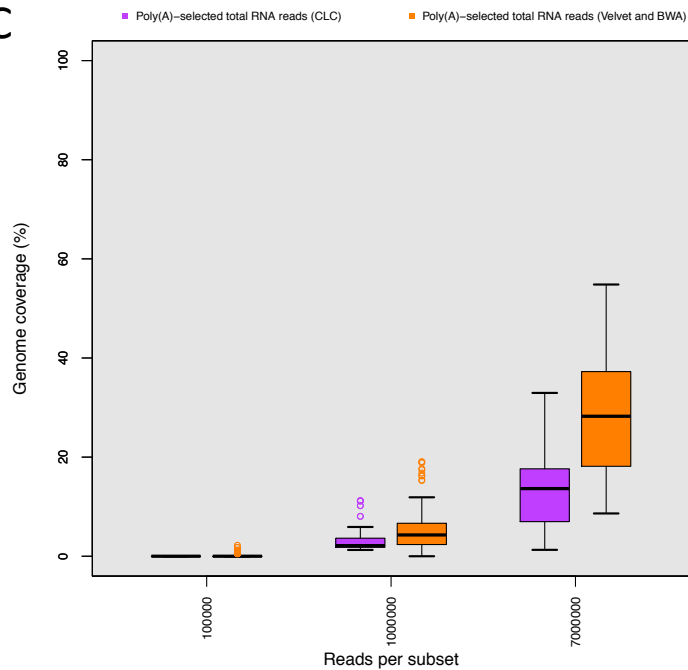**D**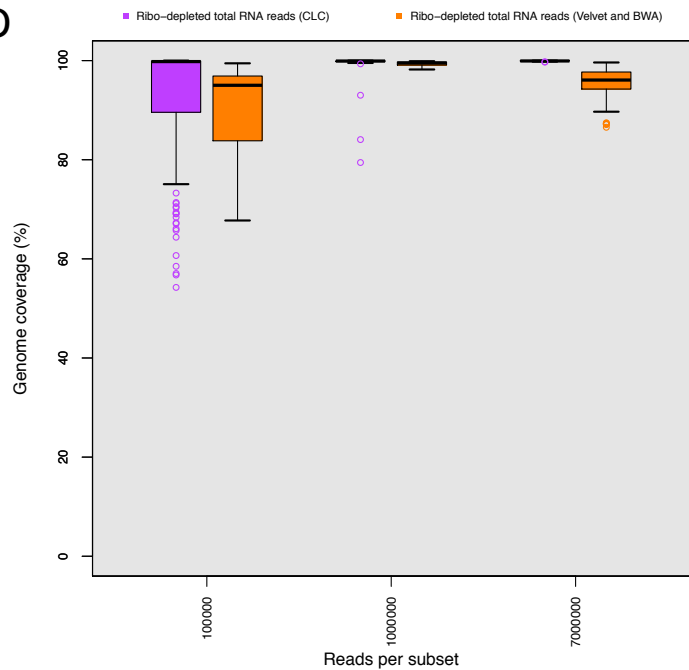

Supplement: Additional file 4: Figure S4. — Average virus genome coverage obtained through de novo assemblies with CLC genomic workbench compared to Velvet. Box-and-whisker plot displaying the data distribution of different subset-sizes of de novo assembled (10 replications) sRNA (A, B), poly(A)-selected (C) or ribo-depleted RNA (D) reads and subsequent mapping of the contigs onto the reference genomes of GLRaV-3 (A, C) and CTV (B, D). For the 7,000,000 reads sRNA subsets, each replication was divided into 1,000,000 reads subsets for de novo assembly after which the contigs were pooled for mapping to the reference genome. (PDF 82 kb) [file 12985_2016_539_MOESM4_ESM.pdf]
